# *N*-(2′-Hydroxyphenyl)-2-Propylpentanamide (HO-AAVPA) Inhibits HDAC1 and Increases the Translocation of HMGB1 Levels in Human Cervical Cancer Cells

**DOI:** 10.3390/ijms21165873

**Published:** 2020-08-16

**Authors:** Yudibeth Sixto-López, Martha Cecilia Rosales-Hernández, Arturo Contis-Montes de Oca, Leticia Guadalupe Fragoso-Morales, Jessica Elena Mendieta-Wejebe, Ana María Correa-Basurto, Edgar Abarca-Rojano, José Correa-Basurto

**Affiliations:** 1 Laboratorio de Diseño y Desarrollo de Nuevos Fármacos e Innovación Biotecnológica), Sección de Estudios de Posgrado e Investigación, Escuela Superior de Medicina, Instituto Politécnico Nacional, Plan de San Luis y Salvador Díaz Mirón s/n, Casco de Santo Tomás, Ciudad de México 11340, Mexico; syudibeth@hotmail.com; 2 Laboratorio de Biofísica y Biocatálisis, Sección de Estudios de Posgrado e Investigación, Escuela Superior de Medicina, Instituto Politécnico Nacional, Plan de San Luis y Salvador Díaz Mirón s/n, Casco de Santo Tomás, Ciudad de México 11340, Mexico; lety.23fm@gmail.com (L.G.F.-M.); jesmenwej@yahoo.com (J.E.M.-W.); anmar_005@yahoo.com.mx (A.M.C.-B.); 3 Laboratorio de Bioquímica, Sección de Estudios de Posgrado e Investigación, Escuela Superior de Medicina, Instituto Politécnico Nacional, Plan de San Luis y Salvador Díaz Mirón s/n, Casco de Santo Tomás, Ciudad de México 11340, Mexico; neocontis@hotmail.com; 4 Laboratorio de Respiración Celular, Sección de Estudios de Posgrado e Investigación, Escuela Superior de Medicina, Instituto Politécnico Nacional, Plan de San Luis y Salvador Díaz Mirón s/n, Casco de Santo Tomás, Ciudad de México 11340, Mexico; rojanoe@yahoo.com

**Keywords:** HO-AAVPA compound, HDAC1 inhibition, HMGB1, SiHa cells, valproic acid

## Abstract

*N*-(2′-hydroxyphenyl)-2-propylpentanamide (HO-AAVPA) is a VPA derivative designed to be a histone deacetylase (HDAC) inhibitor. HO-AAVPA has better antiproliferative effect than VPA in cancer cell lines. Therefore, in this work, the inhibitory effect of HO-AAVPA on HDAC1, HDAC6, and HDAC8 was determined by in silico and in vitro enzymatic assay. Furthermore, its antiproliferative effect on the cervical cancer cell line (SiHa) and the translocation of HMGB1 and ROS production were evaluated. The results showed that HO-AAVPA inhibits HDAC1, which could be related with HMGB1 translocation from the nucleus to the cytoplasm due to HDAC1 being involved in the deacetylation of HMGB1. Furthermore, an increase in ROS production was observed after the treatment with HO-AAVPA, which also could contribute to HMGB1 translocation. Therefore, the results suggest that one of the possible antiproliferative mechanisms of HO-AAVPA is by HDAC1 inhibition which entails HMGB1 translocation and ROS increased levels that could trigger the cell apoptosis.

## 1. Introduction

Cervical cancer (CC) is one of the most frequently diagnosed cancers in women worldwide, and it has a high mortality rate according to the World Health Organization [1,2], being the fourth leading cause of cancer death in females (7.5%) [3]. CC originates from different factors, including sexually transmitted infections with specific human papillomavirus (HPV) types. Therefore, it has been estimated that approximately 5 million people will die from HPV-infection-induced CC over the next two decades, making it necessary to treat CC to decrease mortality [4].

In this sense, some studies have explored histone deacetylases inhibitors (HDACi) for the treatment of cancers due to its cytotoxic effects inducing cell cycle arrest and apoptosis, among other anticancer effects [5,6]. This facts is due to HDACi acts inhibiting the activity of histone deacetylases (HDACs) enzymes which are responsible for deacetylating the ε-amino group on lysine on the tail of proteins, thereby regulating DNA transcription [6]. Zinc-dependent HDACs are classified into three classes: class I (HDAC1, HDAC2, HDAC3, and HDAC8), class II (HDAC4, HDAC5, HDAC6, HDAC7, HDAC9, and HDAC10), and class IV (HDAC11). Additionally, HDAC class III (sirtuins 1-7) utilizes NAD^+^ as a cofactor [7].

HDACi induce cell apoptosis in HPV carrier cells and inhibit cell growth [8,9]. Additionally, some HDACi compounds, such as valproic acid (VPA), trichostatin A (TSA), and sodium butyrate (NaBut) induce the HPV-16 long control region (LCR) transcriptional activity in cervical cell lines [10].

Therefore, HDACi might be used to treat CC due to the advantages of epigenetic regulation of targeted cancer cells [11]. In addition, HDACi are of great importance to treat cancer due to HDACs can remove acetyl groups from various intracellular proteins not only from histones. High mobility group box 1 protein (HMGB1) is mainly located in the nucleus participating in different activities to maintain the nuclear homeostasis, and the DNA repair. This protein can be deacetylates by HDAC enzymes. The acetylation and deacetylation process of HMGB1 is related to the apoptosis process due to in the acetylated form HMGB1 is translocated from the nucleus to the cytosol and also in the extracellular space [12,13]. Depending of its localization and the cell type, HMGB1 can have several functions and also induces several process of cell death [14,15]. In addition, this fact has been associated with immunogenic tumor cell death induced after HDACi administration as a consequence of release of HMGB1 [16].

Therefore, the localization and the effects produced by HMGB1 are related with the HDAC activity which modifies its acetylation status and then its localization [14]. Consequently, HDACi has direct effect on HMGB1 activity due to levels of acetylated HMGB1 increased with a concomitant decrease in HDAC activity, [17,18]. The HDAC1 is the most associated isoform which performs the deacetylation of HMGB1 [18]

In addition, the HMGB1 translocation from the nucleus to the cytoplasm is also regulated by the reactive oxygen species (ROS), for example, hydrogen peroxide (H_2_O_2_) activates the MAPK and NFκB pathways, which produce the release of HMGB1 and also the protein can be oxidased directly on its thiols groups [14].

**Scheme 1:**
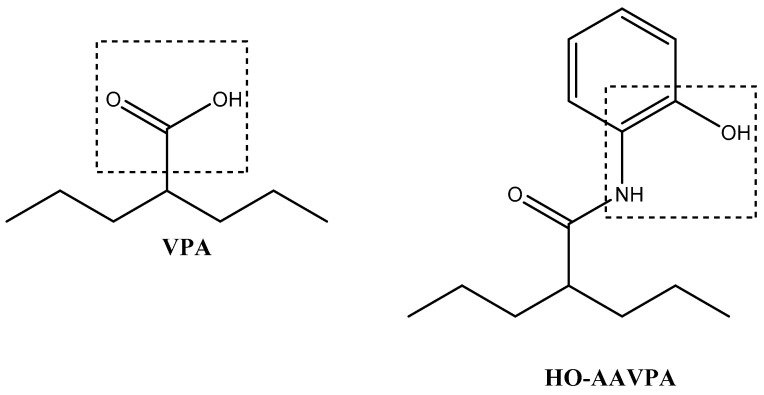
Chemical structures of valproic acid and HO-AAVPA (*N*-(2-hydroxyphenyl)-2-propylpentanamide). HO-AAVPA was designed using valproic acid (VPA) and suberoylanilide hydroxamic acid (SAHA) moieties. The square indicates the chemical moieties which are bound to zinc ions of HDAC enzymes.

*N*-(2-hydroxyphenyl)-2-propylpentanamide (HO-AAVPA) is a VPA derivative designed by in silico studies to target HDAC (Scheme 1), showing better antiproliferative effects than VPA on the rhabdomyosarcoma cell line (A204), cervical cancer cells (HeLa cells), and breast cancer cells (MCF-7, MDA-MB-231, and SKBr3) [2]. Additionally, HO-AAVPA depicted lesser toxicity and teratogenic effects that VPA and also showed better pharmacokinetic properties than VPA in a rat model (half-life for VPA 1 h and for HO-AAVPA 4 h) [19,20,21]. However, the HDAC inhibitory effects of HO-AAVPA have not been experimentally tested, and the mechanism that explains its antiproliferative effect must be elucidated. Therefore, this study evaluated the HO-AAVPA affinity for class I HDACs, namely, HDAC1, HDAC8, and HDAC6, using in vitro and in silico models, due to having been reported that HDAC 1 and 8 are associated with HMGB1 deacetylation [22]. Further, HO-AAVPA was evaluated in the SiHa cervical cancer cell line to measure its cytotoxicity and to identified the HMGB1 localization; after the treatments, the levels of superoxide anions (O_2_^−·^) were measured.

## 2. Results

### 2.1. HO-AAVPA Has Better Affinity and Binding Mode on HDAC1 by Molecular Docking

Molecular docking of HO-AAVPA on HDAC 1, 8, and 6 was done and the binding and interactions profile was analyzed. As is showed in Table 1, according to molecular docking results, the affinity of HO-AAVPA for HDACs was found to be in the following order: HDAC1 > HDAC8 > HDAC6_DD1 > HDAC6_DD2.

**Figure 1:**
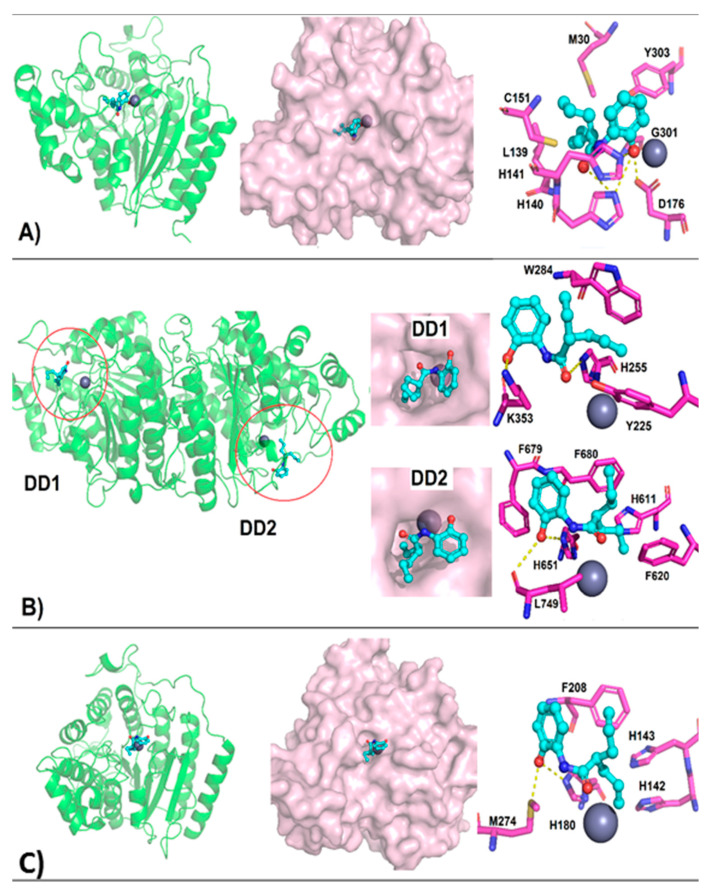
Molecular docking of HO-AAVPA with different HDAC isoforms. (**A**) HDAC1, (**B**) HDAC6, and (**C**) HDAC8. On the left side of the panel, the green ribbon represents the HDAC interacting with HO-AAVPA depicted as cyan ball and stick; in the center of the panel, the pink surface represents HDAC, and the ligand is shown as cyan ball and stick. On the right side, the HDAC residues that interact with HO-AAVPA are depicted with a gray ball and stick representation. Zn^+2^ is depicted as a gray sphere, and on the right side, the map of the interactions between HDACs and the ligands are depicted and explained in the text (Figure built with PyMOL v.099 and Discovery Studio v 16.1).

The binding mode generated revealed that HO-AAVPA reached the catalytic site only of HDAC1, where the oxygen atom of the phenyl hydroxyl group is coordinated with Zn^+2^ atoms in a monodentate manner such that the hydroxy-phenyl moiety acted as a zinc binding group (ZBG). Additionally, the hydroxyl group formed a hydrogen bond with D176, whereas the phenyl group makes π-π interactions with Y303 and H141 in the catalytic site. The oxygen of the carbonyl group formed a hydrogen bond with the side chain amine group H140 (Figure 1A). In addition, the amide and the aliphatic portion of HO-AAVPA were inserted into the 14-Å tunnel.

In HDAC6, the aliphatic portions of HO-AAVPA were inserted into the catalytic tunnel rich in aromatic residues located at the catalytic site from both HDAC6 domains, DD1 and DD2. In DD1, the aliphatic moiety of HO-AAVPA interacted hydrophobically with the residues lining the catalytic site, such as W284, H255, and Y225. In addition, there are hydrogen bonds between hydroxyl of the aromatic ring with the oxygen of the amide group of the K353 backbone, while the amide carbonyl group of HO-AAVPA formed a hydrogen bond with side chain of H255. Y225 and K353 that are important residues for catalytic activity by functioning as gatekeepers [23]. The aliphatic chain of HO-AAVPA reached DD2, interacting with the hydrophobic residues located in the catalytic tunnel (H611, F620, and F680); the phenyl group of the HO-AAVPA interacted via π-π interactions with the side chain of F679 and the hydroxyl group formed a hydrogen bond with L749, a residue that belongs to the cap region [24]. (Figure 1B).

HDAC8 accommodated the HO-AAVPA aliphatic chain, enabling it to reach the catalytic tunnel and established π-alkyl interactions with H180, H142, H143, and F208, while the oxygen of the hydroxyl group of the HO-AAVPA established hydrogen bonds with side chains of H180 and M274, whereas there are π-π interactions with the side chain of the Y306 (Figure 1C).

### 2.2. HO-AAVPA Has Better Inhibitory Activity on HDAC1 Than HDAC6 and HDAC8

**Figure 2:**
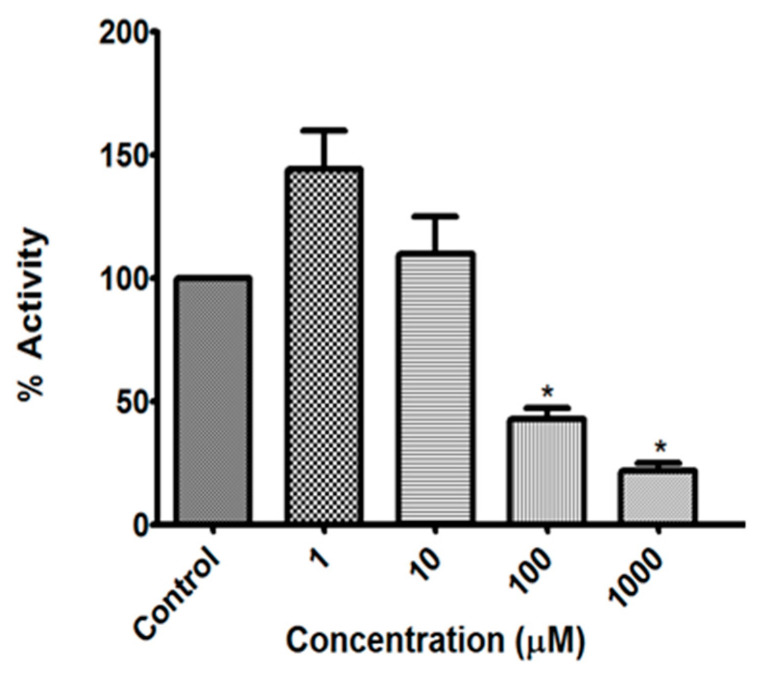
HDAC1 inhibition by HO-AAVPA. The inhibitory effect observed for HDAC1 with HO-AAVPA was dependent of the concentration. The values shown are the means of the % activity ± SE of duplicate measurements from two independent experiments. The significant difference is showed by * *p* < 0.05 obtained from one-way ANOVA test with Dunnett post-test.

The inhibitory activity of HO-AAVPA was evaluated in vitro using recombinant class I HDAC1 and HDAC8 and class II HDAC6. As is depicted in Figure 2, HO-AAVPA has inhibitory activity on HDAC1 due to at 100 μM, the percent of activity was less than 50%, having significant difference with the control (* *p* < 0.05). However, HO-AAVPA had no inhibitory activity on HDAC6 and HDAC8 (Table 2). The IC_50_ of HO-AAVPA on HDAC1 was determined being IC_50_ = 153 μM (Table 2). Furthermore, trichostatin A (TSA) was employed as positive control, observing that this has similar IC_50_ values on all HDACs evaluated (Table 2).

### 2.3. HO-AAVPA Has Antiprolifetarive Effects on SiHa Cells

**Figure 3:**
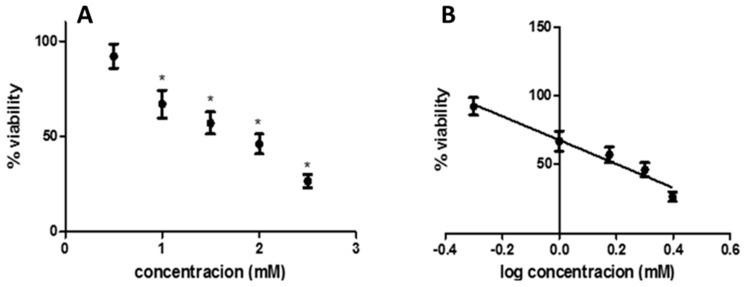
Viability of SiHa cells treated with HO-AAVPA evaluated by MTT assay. (**A**) SiHa cells treated with HO-AAVPA, a decrease in the cell viability was observed since 1 mM having all concentrations tested significant difference vs. the concentration of 0.5 mM. (**B**) The IC_50_ of 1.6 mM for HO-AAVPA in SiHa cells was obtained by linear regression from the log of the concentrations vs. % viability. The experiments were performed in triplicate with *n* = 8 for each concentration. Data are presented as the means ± SE. * *p* < 0.05 obtained from one-Way ANOVA analysis and Dunnet as post-test.

The antiproliferative effect of HO-AAVPA on SiHa cells was measured after 48 h of treatment (Figure 3A). HO-AAVPA caused cell death at concentrations of 1 mM and greater in a concentration-dependent manner; at 2.5 mM, approximately 25% viability was observed, Figure 3A showed significant difference between the concentrations evaluated with the concentration at 0.05 mM. The IC_50_ of HO-AAVPA was obtained being of 1.6 mM (Figure 3B).

### 2.4. HMGB1 Localization in SiHa Cells Treated with VPA and HO-AAVPA

**Figure 4:**
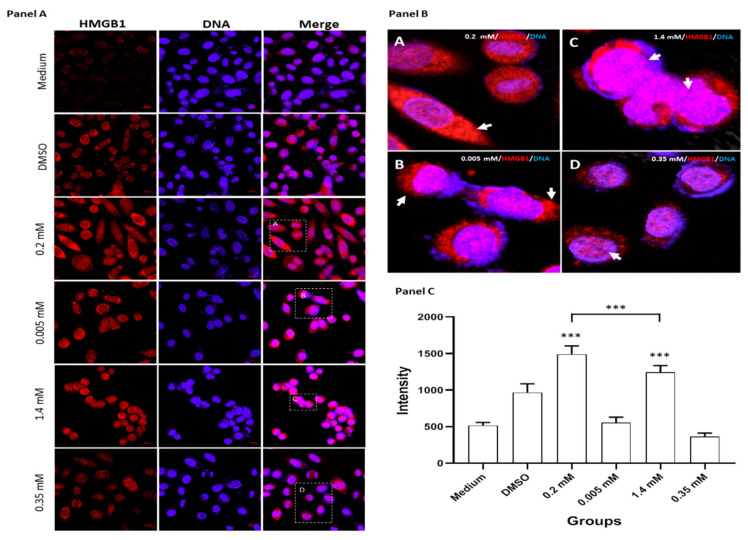
Intracellular location of HMGB1 in SiHa cells in response to treatment with VPA and HO-AAVPA. SiHa cells were stimulated with VPA at 1.4 and 0.35 mM, HO-AAVPA at 0.2 and 0.005 mM for 12 h. In Panel (**A**), is observed HMGB1 which was immunostained with anti-HMGB1 Ab and Alexa 647–conjugated secondary Ab (red) and DAPI to observe the DNA (blue) and the merge to observed the colocalization of HMGB1. In Panel (**B**), is shown the amplified images of the boxes shown in Panel (**A**) labeled in squares. The figure (**A**,**B**) in the Panel (**B**) are showing HMGB1/DNA in presence of HO-AAVPA at 0.2 and 0.005 mM, respectively. The figure (**C**,**D**) are showing HMGB1/DNA in presence of VPA at 1.4 and 0.35 mM, respectively. It is observed that the cells treated with HO-AAVPA have greater intensity of translocation of HMGB1 from the nucleus to the cytoplasm (white arrows), while for VPA HMGB1, was observed predominantly in the nucleus. In Panel (**C**), is shown the analyses of the fluorescent intensity, where it is observed that the intensity for HMGB1 is statistically higher in cells stimulated with HO-AAVPA (0.2 mM) with respect to VPA (1.4 mM) (*p* < 0.001) and both with respect to the vehicle group (*p* < 0.001). The graphic shows two independent experiments and the average of 10 cells in the visual field in microscopy (400×). Data are presented as the mean ± SE of intensity. *** *p* < 0.001 vs. DMSO vehicle.

HMGB1 levels in SiHa cells were evaluated by confocal microscopy at two different concentrations of either HO-AAVPA (0.2 and 0.005 mM) or VPA (1.4 and 0.35 mM), it is important to mention that these concentrations were used due to being less than their IC_50_ values. For HO-AAVPA (1.6 mM), although in this study we do not have a IC_50_ for VPA in SiHa cells, in other studies it has been reported for cytotoxic effects of VPA at a concentration near to 3 mM, and the most usual VPA concentration that did not produce significant effect on the proliferation of cervical cancer cells is 1 mM [25]. The results in Figure 4, Panel A, showed the immunostaining of HMGB1 (red) which is present in all samples, although in less amount in the sample with medium and increase with the treatments, also in those treated with the vehicle (DMSO 0.1%). To observe the nucleus, the samples were treated with DAPI (blue) as is observed in all samples. After, to see the localization of HMGB1 in all treatments, a merge was done. The localization of HMGB1 is shown more clearly in Figure 4B, where it is possible to observe in Figure 4A that the treatment of the cells with HO-AAVPA at 0.2 mM produce high amount of HMGB1 in the cytoplasm than in the nucleus, contrary to that observed with VPA at 1.4 mM where HMGB1 is expressed in a larger amount in the nucleus (Figure 4C, Panel B, indicated with white arrows). Employing the low concentrations for both compounds, the same effect in the HMGB1 translocation was observed, induced HO-AAVPA more HMGB1 in the cytoplasm than VPA (Figure 4B,D, indicated with white arrows). In addition, HMGB1 translocation from the nucleus to the cytoplasm was observed and was attributed to the presence of the HO-AAVPA. Furthermore, is possible to observe that the cell morphology changes from spindle to spherical shaped, which is due to the compound and also to the vehicle, being less in the vehicle and in the treatment with HO-AAVPA at 0.2 mM.

The fluorescence intensity of the control group (medium or DMSO vehicle) indicated that the fluorescence intensity for HMGB1 was minimal in the medium and increase with DMSO. However, the increase in the fluorescence was more in HO-AAVPA 0.2 mM and VPA 1.4 mM, having statically significance with the vehicle (*** *p* < 0.001), Figure 4C.

### 2.5. HO-AAVPA Increase the ROS Levels (O_2_^−·^) in the SiHa Cells

**Figure 5:**
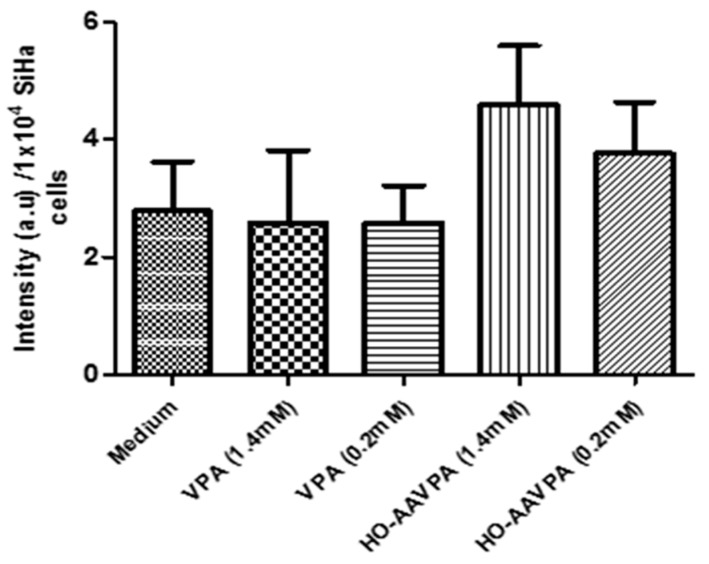
Measurement of ROS (O_2_^−·^) levels by electronic paramagnetic resonance. SiHa cells were treated for 48 h with VPA or HO-AAVPA at two different concentrations: 1.4 and 0.2 mM. The intensity of the EPR signal is proportional to the ROS production and it was plotted against each one of the treatments. The values shown are the means ± SE of duplicate measurements from two independent experiments. No significant difference was obtained by one-Way ANOVA analysis and Dunnet as post-test comparing all treatment with the medium sample.

Superoxide anion (O_2_^−·^) levels were measured in cells pretreated with VPA or HO-AAVPA at two concentrations of 0.2 and 1.4 mM for 48 h. After 48 h of VPA treatment, the cells did not have a significant difference in ROS production regardless of the concentrations administered. However, HO-AAVPA caused an increase in ROS production that was directly dependent on the concentration. ROS production was greater at 1.4 mM and slightly lower, but still higher than the control, at 0.2 mM (Figure 5).

## 3. Discussion

HO-AAVPA is a synthetic compound obtained by a drug rationally designed using in silico tools on the basis that valproic acid (VPA) and suberoylanilide hydroxamic acid (SAHA) moieties are pharmacophores that can inhibit HDAC [2]. The action mechanism of HO-AAVPA in promoting cell death had not been elucidated. There is an initial attempt in which HO-AAVPA was found to increase the levels of acetylated HMGB1 in HeLa cells, a finding that may have been the result of HDAC inhibitory effects [26].

Therefore, in this study, molecular docking studies of HO-AAVPA on HDAC1, HDAC8, and HDAC6 were performed. The docking studies showed the binding free energies that reflect the following order of affinity: HDAC1 > HDAC8 > HDAC6-DD1 > HDAC6-DD2, a finding in agreement with the experimental results described, indicating that HO-AAVPA had the strongest inhibitory effect on HDAC1. These results suggest that HO-AAVPA is not a pan-HDAC inhibitor as VPA which inhibits HDAC classes I, IIa and IIb (HDAC6) [22,27].

The docking studies suggest that HO-AAVPA could block HDAC1 in a dual manner. This, because, the amide and aliphatic moieties of HO-AAVPA are inserted into the 14-Å tunnel, which is postulated to be an acetate release tunnel and also HO-AAVPA recognizes the zinc-binding site that can inhibit the HDAC1 catalytic activity [28,29]. Potent selective HDAC1/2 inhibitors share this binding pose, and it has been postulated that it is the reason for the selectivity of these compounds toward class I HDACs [28,30]. The aliphatic chain of HO-AAVPA establishes hydrophobic interactions with residues belonging to the 14-Å channel, such as M30, L139, and C151 [28]. In addition, the in silico evaluation of HO-AAVPA interacting with HDAC8 indicates that the ligand did not reach the protein catalytic site and that it was inserted in an inverted manner into the catalytic tunnel.

Therefore, we tested the in vitro inhibitory activity of HO-AAVPA against HDAC1, HDAC8, and HDAC6. Our results indicated that HO-AAVPA inhibits HDAC1 (IC_50_ = 153.78 μM), while no inhibition was observed for HDAC6 and HDAC8. Therefore, these results correlated with docking results showing that HO-AAVPA is capable to be accommodate at catalytic site of HDAC1 enzyme, suggesting that it affects the deacetylation activity. Thus, it explains the increase acetylation of HMGB1 due to HDAC1 inhibition, then, the HO-AAVPA favored the acetylate HMGB1 [26,31,32,33,34]. All these processes, such as HDAC1 inhibition as well HMGB1 acetylation, could be related with the cytotoxic effect observed for HO-AAVPA (IC50 = 1.6 mM at 48 h). Therefore, the chemical structural modification on HO-AAVPA in relation to VPA, improved its antiproliferative effect and although it is still not at pharmacological concentration for this kind of cells, was possible to obtain better results than with VPA [25].

These findings (in silico and in vitro) are in agreement with previous studies that linked the increase in acetylated HMGB1 with HDAC1 inhibition [18,31]. Therefore, the HMGB1 localization in the cells was determinated before and after VPA and HO-AAVPA treatment, a significant increase of HMGB1 was detected in SiHa cells treated with HO-AAVPA (0.2 mM) and VPA (1.4 mM). In addition, HMGB1 was translocated from the nucleus to the cytoplasm in the cells treated with HO-AAVPA, suggesting that HMGB1 is acetylated and it cannot back to the nucleus, therefore, acetylated HMGB1 could exit from the cell and participate in cell death [13,15]. In addition, an increase of HMGB1 in the cytoplasm is in agreement with the amount of O_2_^−·^ produced at each concentration, as was demonstrated in HeLa cells in which after HO-AAVPA treatment induced oxidative stress and also the levels of acetylated HMGB1 were increased, and these results were also linked with the possible HDAC1 inhibition [26,31].

The O_2_^−·^ levels in the SiHa cells increased in the presence of HO-AAVPA at 1.4 mM, which could be related to increased oxidative stress and possibly more oxidized HMGB1 increasing its cytotoxicity and inducing apoptosis by the caspase-9/-3 intrinsic pathway [35]. Furthermore, an increase in O_2_^−·^ levels in the presence of HO-AAVPA could be related to HDAC inhibition due a relationship between HDAC inhibition and ROS has been reported [17]. Showing these results, a correlation between higher intracellular ROS levels and high cell death sensitivity is due to the treatment [36,37]. The relationship between ROS and HMGB1 has been widely documented, with an increase in HMGB1 levels as well as an increase in cytoplasmic translocation from the nucleus as consequences of ROS overproduction [14,38]. Therefore, more studies employing HO-AAVPA could be done to demonstrated if the cells after the treatment with the compound death by apoptosis, induced by HDAC1 inhibition, by HMGB1 translocation or by ROS production or as consequence each other. In addition, to determine if HMGB1 is extracellular or is oxidized and participate in the induction of apoptosis. 

## 4. Materials and Methods

### 4.1. Molecular Docking

The chemical structure of HO-AAVPA was drawn in Gaussian View 5.0 [39], and it was further optimized in a vacuum using Gaussian 09 [40] with the Austin. 

Model 1 (AM1) semi empirical method. The output files were converted to Protein Databank (PDB) format using Gaussian View 5.0 [38]. Then, HO-AAVPA was prepared for docking in AutoDock Tools 1.5.6 [41] and only polar hydrogen atoms, flexible bonds, and partial Gasteiger atomic charges were assigned for the docking simulations [42]. Proteins were retrieved from https://www.rcsb.org with the following PDB codes: 4BKX for HDAC1 and 3F07 for HDAC8, [43]. Additionally, HDAC6 was taken from a model that included both catalytic sites of HDAC6 (DD1 and DD2) [44]. The HDACs were prepared using AutoDock Tools 1.5.6 [40] with polar hydrogen atoms and Kollman charges added [45].

Molecular docking was performed using AutoDock 4.2 [24], centering the grid box on Zn^+2^, with a dimension of 60 Å^3^ and grid spacing of 0.375 Å^3^. A Lamarckian genetic algorithm was used as a scoring sample with a randomized population of 100 individuals and energy evaluations of 1 × 10^7^; further; 100 runs were performed. To select the best binding pose, cluster analysis with the lowest free energy values was selected. Docking results were analyzed using AutoDock Tools 1.5.6 [41] and Discovery Studio 2017 R2 [46]. The figures were further processed with PyMOL v.099 [47].

### 4.2. Chemical Substance and Compounds

VPA (sodium valproate) was purchased from Sigma-Aldrich Co., Toluca City, Mexico. HO-AAVPA was synthesized in our laboratory following a synthesis route published previously [2]. TSA was purchased from Enzo Life Sciences (Farmingdale, NY, USA).

### 4.3. Biological Procedure

#### 4.3.1. HDAC Inhibitory Activity

Fluor de LYS HDAC1 (BML-AK511), HDAC6 (BML-AK516), and HDAC8 (BML-AK518) drug discovery kits were purchased from Enzo Life Sciences (Farmingdale, distribute in Mexico City by Industrias Bioselec S.A. de C.V., Mexico City, Mexico) to assay the HDAC inhibitory effect of HO-AAVPA at 1, 10, 100, and 1000 μM. For the assays, each HDAC was incubated at different concentrations and temperatures due to enzyme kits conditions. The HDAC1 was incubated at 250 ng/well at 37 °C with 5 µM of substrate; HDAC6 at 400 ng/well at 37 °C with 12 µM of substrate, and HDAC8 was incubated at 1 U/well at 30 °C with 50 μM of the substrate. The procedure was done taking into account the recommendations provided in the kit. The fluorescence was measured in a fluorometer (LS 55, Perkin Elmer, Agilent, Santa Clara, CA, USA) with excitation at 360 nm and emission at 460 nm. Trichostatin A (TSA) was evaluated as a positive control. IC_50_ values were determined by linear regression fit using GraphPad Prism 5 software (San Diego, CA, USA).

#### 4.3.2. Cell Culture

SiHa cells were kindly donated by Dr. Nicolás Villegas Sepulveda from the Department of Molecular Biomedicine, CINVESTAV, México. SiHa cells were cultured in Dulbecco’s modified Eagle’s medium (DMEM Gibco, Life Technologies, Invitrogen, Sigma-Aldrich Co., Toluca City Mexico), supplemented with 10% fetal bovine serum (FBS, Biowest, Kansas City, MO, USA) and antibiotic and antimycotic agents (Gibco, Fisher Scientific UK Ltd, Loughborough, UK). The cultures were kept at 37 °C in a humidified atmosphere with 5% CO_2_.

#### 4.3.3. Cell Viability by MTT Assay

After seeding 10 × 10^3^ cells per well in transparent 96-well plates, the cells were incubated for 24 h in DMEM medium with FBS. Then, the cells were incubated with different concentrations of HO-AAVPA (0.5, 1.0, 1.5, 2.0, and 2.5 mM) and VPA (0.625, 1.25, 2.5, 5.0, and 7.5 mM). After 48 h of incubation, the supernatant was removed. Then, 20 μL of MTT solution (0.5 mg/mL) in buffer was added, and the cells were incubated at 37 °C for 4 h. After the MTT solution was removed from all the wells, the formazan salts were solubilized with 100 μL of DMSO, and the absorbance of each well was read at 550 nm on a spectrophotometer (Multiskan EX Microplate Photometer, Thermo Scientific, Mexico).

#### 4.3.4. Localization of HMGB1 by Confocal Microscopy

A total of 20 × 10^3^ cells per well were added to 24-well plates and incubated for 24 h in DMEM with FBS. Then, the cells were washed with phosphate buffered saline (PBS 1X). The groups received the following treatments: VPA at 1.4 and 0.35 mM, HO-AAVPA at 0.2 and 0.005 mM, vehicle (DMSO 0.1%), and incubated for 12 h. After incubation, the cells were fixed with paraformaldehyde (PFA 4%) at 37 °C for 20 min and permeabilized (PBS Tween-Triton 0.5%) for 5 min. Subsequently, the cells were washed and the primary antibody, anti-HMGB1 (GTX101277, GeneTex, Alton Pkwy Irvine, CA, USA), was added at a ratio of 1:1000 and incubated overnight. The secondary antibody, donkey anti-rabbit IgG H&L (Alexa Fluor 647, Thermo Fisher Scientific, México City, Mexico), was added at a ratio of 1:1000 and incubated at 4 °C for 4 h. Finally, the slides were washed with PBS and mounted with Vectashield (Mounting Laboratories, Inc. Burlingame, CA, USA).

The images were collected and analyzed with an Axioskop 2 mot plus confocal fluorescence microscope (Carl Zeiss, Mexico City, Mexico) at EC Plan-Neofluar 20×–40×/0.5 Ph2: LP 650, LP 505.

#### 4.3.5. Quantification of Superoxide Anion by Electronic Paramagnetic Resonance

The SiHa cells (500 × 10^3^) were seeded in a 22 m^2^ dish and kept at 37 °C in a humidified atmosphere with 5% CO_2_ until they reached 70% confluence. After, the cells were treated with two different concentrations of VPA and HO-AAVPA (1.4 and 0.2 mM) and maintained for 48 h, the experiment was performed in triplicate and used as a control for data comparison with the SiHa cells treated only with medium. Next, the cells were trypsinized and centrifuged at 3000 rpm for 10 min, and the cell pellet was treated with the radical scavenger CM-H (1 hydroxy-3-methoxycarbonyl-2,2, 5,5-tetramethyl pyrrolidine) diluted in Krebs buffer (99 mM NaCl, 4.69 mM KCl, 2.5 mM CaCl_2_ × 2H_2_O, 1.2 mM MgSO_4_ × 7 H_2_O, 2.5 mM NaHCO_3_, 1.03 mM KH_2_PO_4_, 5.6 mM D-Glucose, and 20 mM Na-HEPES) with sodium diethyldithiocarbamate trihydrate (DETEC; 5 μM) and deferoxamine methane sulfonate salt (DF 25 μM) at a 89:3:8% *v/v* ratio of Krebs buffer: DETEC:DF. The stock solution of CMH (10 mM) was used to dilute each sample to 0.5 mM. The CM-H solution and the sample preparation were processed inside a nitrogen atmosphere chamber. The samples containing CM-H were incubated for 30 min at 37 °C with constant shaking. After the samples were centrifuged at 3000 rpm for 15 min, 50 µL of the supernatant was taken up with capillaries (Corning, Glendale, CA, USA) inside the nitrogen chamber, and each of the capillaries were sealed on both sides to determine the EPR measurements.

The measurements of O_2_^−·^ formation were performed in a Bruker e-scan spectrometer with the following parameters recorded: magnetic field 3490 G, window: 60 G, amplitude: 1.5 G, gain: 56.4, power: 0.0155 mW; and frequency: 9.743 GHz. WinEPR Acquisition software was used to analyze the results. The intensity of each sample was measured and normalized to the cell number for each sample. Finally, the result was expressed as the intensity for 10,000 cells.

### 4.4. Statistical Analysis

The results are presented as the mean ± standard error SE. Data were analyzed by one-way ANOVA followed by Dunnett test to compare all columns with the control to determine statistically significant differences between the groups indicated by * *p* < 0.05. All analyses and graphs were realized using GraphPad Prism version 5.00 software. Inc., San Diego, CA, USA.

## 5. Conclusions

HO-AAVPA compound is capable to inhibit HDAC1 and consequently influence in the HMGB1 translocation in the cells, also mediated by the ROS production, processes that can be related with the cell death. Furthermore, these findings indicates that this compound could be a selective HDAC1 inhibitor, possibly due to the benzamide pharmacophore that is shared among HDAC class I inhibitors.

## Figures and Tables

**Table 1:** Binding free energy (ΔG) and theoretical Kd values, predicted from the interaction of HO-AAVPA with HDAC1, HDAC6, and HDAC8 by molecular docking analyses.

**HDAC Isoform**	ΔG (kcal/mol)	Kd (µM)
**HDAC1**	−5.08	188.93
**HDAC6-DD1**	−4.65	387.40
**HDAC6-DD2**	−4.45	547.71
**HDAC8**	−5.07	191.86

**Table 2:** IC_50_ values of HO-AAVPA on HDAC1, HDAC6, and HDAC8.

Compounds	IC_50_ (µM) *		
	HDAC1	HDAC6	HDAC8
HO-AAVPA	153.78 ± 25.18	>1000	>1000
TSA	0.00761 ± 0.00172	0.00972 ± 0.00002	0.00989 ± 0.00379

* The values shown are the means ± SE of duplicate measurements from two independent experiments. IC_50_ values were determined by linear regression fit using GraphPad Prism 5 software.

## Abbreviations

HO-AAVPA	N-(2′-hydroxyphenyl)-2-propylpentanamide
SAHA	Suberoylanilide hydroxamic acid
HDAC	Histone deacetylase
HMGB1	High mobility group box 1 protein
ROS	Reactive oxygen species
CC	Cervical cancer
HVP	Human papillomavirus
HDACI	Histone deacetylases inhibitors
TSA	Trichostatin A
NABUT	Sodium butyrate
LCR	Long control region
H2O2	Hydrogen peroxide
O2.-	Superoxide anions
ZBG	Zinc binding group
VPA	Valproic acid
PBS	Phosphate buffered saline
PFA	Paraformaldehyde
DETEC	Diethyldithiocarbamate trihydrate
DF	Deferoxamine methane sulfonate salt
CM-H	hydroxy-3-methoxycarbonyl-2,2, 5,5-tetramethyl pyrrolidine
